# Linear association of compound dietary antioxidant index with hyperlipidemia: a cross-sectional study

**DOI:** 10.3389/fnut.2024.1365580

**Published:** 2024-02-29

**Authors:** Haoran Zhou, Tianshu Li, Jie Li, Dongdong Zheng, Jie Yang, Xin Zhuang

**Affiliations:** ^1^Shandong University of Traditional Chinese Medicine, Jinan, China; ^2^Department of Cardiology, The Affiliated Hospital of Shandong University of Traditional Chinese Medicine, Jinan, China

**Keywords:** CDAI, hyperlipidemia, relationship, a cross-sectional study, NHANES

## Abstract

**Background:**

There is growing evidence that antioxidant-rich diets may prevent hyperlipidemia. However, the relationship between the Composite Dietary Antioxidant Index (CDAI) and hyperlipidemia is unclear. The CDAI is a composite score reflecting the antioxidant content of an individual’s diet, and this study aimed to investigate the relationship between CDAI and hyperlipidemia.

**Methods:**

The study used the 2003–2018 National Health and Nutrition Examination Survey (NHANES) database for cross-sectional analyses and included 27,626 participants aged 20 years and older. The CDAI, which includes vitamins A, C, and E, zinc, selenium, and carotenoids, was calculated based on dietary intake reported in a 24-h recall interview. Hyperlipidemia was defined by the National Cholesterol Education Program (NCEP). Covariates included age, sex, race, education, marriage, household poverty-to-income ratio (PIR), glomerular filtration rate (eGFR), body mass index (BMI), energy, carbohydrates, total fat, cholesterol, smoking, alcohol consumption, hypertension, diabetes mellitus, coronary heart disease, and lipid-lowering medications. The association between CDAI and hyperlipidemia was explored through multiple logistic regression analyses and smoothed curve fitting. We also performed subgroup analyses and interaction tests to verify the relationship’s stability.

**Results:**

After adjusting for potential confounders, CDAI was negatively associated with the risk of developing hyperlipidemia (OR 0.98, 95% CI 0.96–0.99, *p* < 0.01). The results of weighted regression models stratified by quartiles of CDAI (−8.664 ≤ Q1 ≤ −2.209, −2.209 < Q2 ≤ −0.002, −0.002 < Q3 ≤ 2.774, 2.774 < Q4 ≤ 124.284), fully adjusted for confounding variables, indicated that compared with the bottom quartile (Q1) of the CDAI, Q2, Q3, and Q4 of participants had a lower advantage ratio (Q2: OR 0.91, 95% CI 0.78–1.06, *p* < 0.21; Q3: OR 0.85, 95% CI 0.73–1.00, *p* < 0.05; and Q4: OR 0.77, 95% CI 0.64–0.94, *p* < 0.01), which was confirmed by a test for trend (*p* < 0.05). Smoothed curve fit analysis showed linearity (p for non-linear = 0.0912). In summary, there is a linear negative relationship between CDAI and the risk of developing hyperlipidemia. Subgroup analyses by age, sex, ethnicity, education level, marriage, tobacco status, alcoholic drinking, body mass index (BMI), hypertension, and diabetes did not indicate strong interactions.

**Conclusion:**

In this large cross-sectional study, there was a linear negative association between CDAI and hyperlipidemia among US adults. Therefore increase antioxidant rich foods in your life as a prevention of hyperlipidemia.

## 1 Introduction

Hyperlipidemia is a metabolic disorder in which there is an abnormally high level of lipids in the blood and is clinically classified as hypercholesterolemia, hypertriglyceridemia, mixed hyperlipidemia, and low HDL cholesterolemia, depending on the type of elevated lipoprotein (1, 2). Hyperlipidemia has been a widespread concern in Europe, America, and other developing countries. According to epidemiologic surveys, it was found that in the United States, about 12% of adults ≥20 years of age had total cholesterol levels higher than 240 mg/dL, and about 17% had high-density lipoprotein (HDL) cholesterol levels <40 mg/dL between 2015 and 2018 (3). Hyperlipidemia is one of the risk factors for several vascular diseases (4). Thus, it increases the risk of disease in the elderly (5). The prevalence of hyperlipidemia continues to grow as the world faces an increasingly aging population (6). Hyperlipidemia is associated with a variety of diseases such as stroke, diabetes, coronary heart disease, and other chronic diseases (7), severely reducing the quality of life, shortening the lifespan of the patients, and causing a substantial economic burden. Based on this fact, finding effective treatments to address this problem is essential.

Current research has identified the key to oxidative stress as reactive oxygen species (ROS), and the overproduction of ROS has been linked to pathological diseases such as obesity, insulin resistance, hyperglycemia, chronic inflammation and dyslipidemia (8–10). During physiological circumstances, ROS are readily modulated by counter-oxidants. However, counter-oxidants can be obtained *in vivo* and *in vitro* (11). When antioxidant deficiencies and malnutrition result in the body being more susceptible to oxidative stress, thus improving the risk of an adverse reaction (12). There is evidence of an association between hyperlipidemia and oxidative stress (13). Vitamin A (VA) as a dietary antioxidant, Wang et al. (14). In an intervention experiment in diabetic adipose rats, administration of VA for 8 weeks was able to improve fat metabolism to reduce hyperlipidemia. Vitamin E (VE) derives its antioxidant activity mainly from α-tocopherol and γ-tocopherol. In a study in children and adolescents, administration of micronutrients VE and VA was shown to reduce blood lipids through antioxidant properties (15). Administration of zinc alone in type 2 diabetic rats improved lipid levels (16), in addition to dietary zinc reducing oxidative stress (17). It is apparent that numerous studies have confirmed the effect of single antioxidants on hyperlipidemia, but the relationship between combined dietary antioxidant intake and hyperlipidemia still needs to be explored.

In recent years, the role of antioxidants has become prominent in global dietary patterns, and the impact of total nutritional antioxidant capacity on health has become increasingly common in all sectors (18). Dietary antioxidant supplementation through consuming fruits, vegetables, whole grains, nuts, and legumes has been suggested (19), and the intake of adequate antioxidants may help reduce the burden of oxidative stress (20). CDAI was proposed by Wright et al. (21) as a composite score reflecting antioxidant capacity of one’s diet, which includes vitamins A, C, and E, zinc, selenium, and carotenoids. Previous studies have found that CDAI improves heart failure, hypertension, depression, and atherosclerotic cardiovascular disease and reduces the risk of morbidity (18, 22–24). However, there are no studies on the relationship between CDAI and hyperlipidemia.

In this study, we explored the association between CDAI and hyperlipidemia for the first time using a large-sample cross-sectional design. Based on previous studies, it was hypothesized that there may be a negative association between CDAI and hyperlipidemia, intending to prevent hyperlipidemia and reduce the risk of its onset through diet, with important clinical implications.

## 2 Materials and methods

### 2.1 Study participants

**Figure 1:**
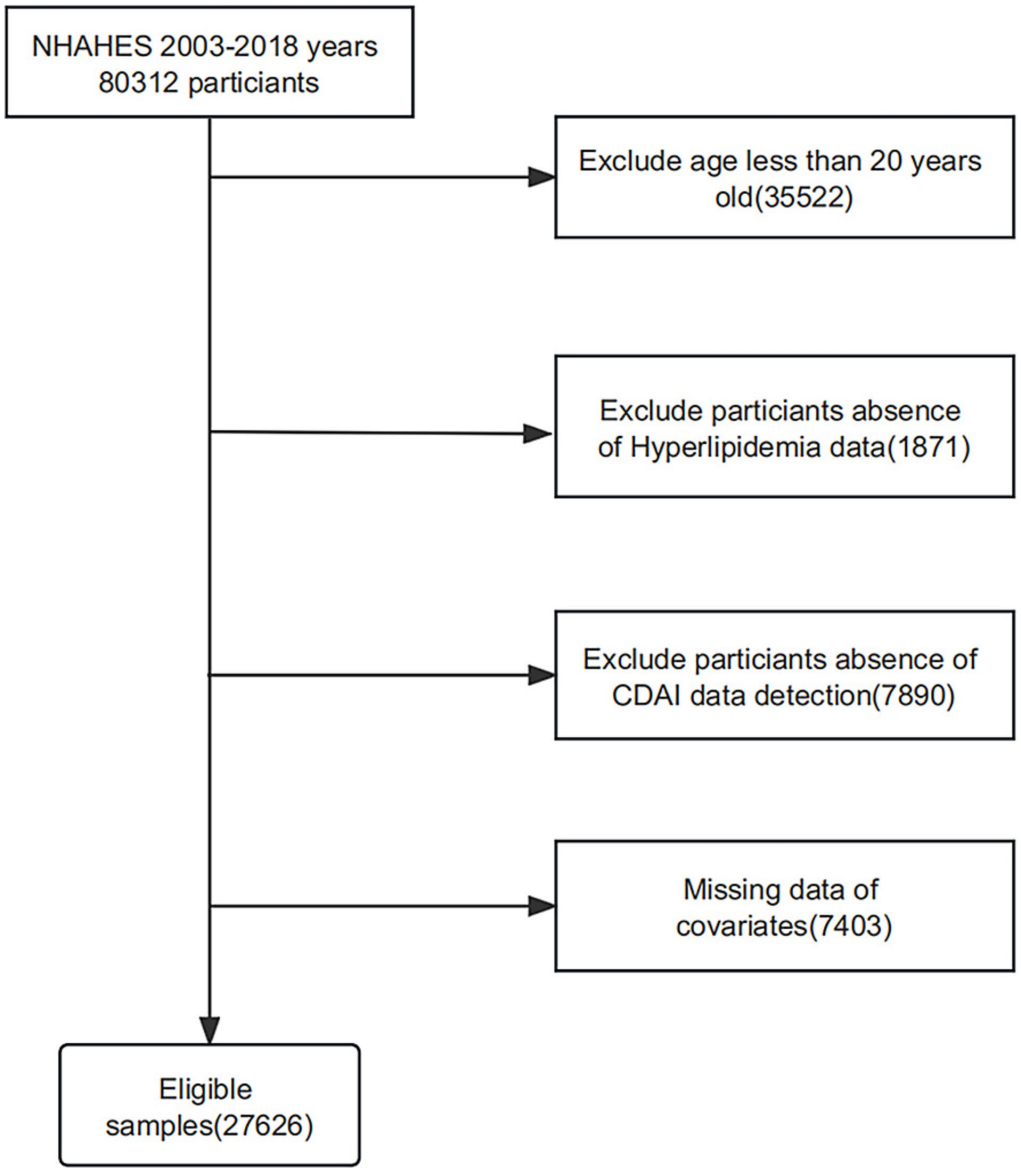
Flowchart depicting participant selection in the study.

The National Health and Nutrition Examination Survey (NHANES) is a United States population census intended to accurately estimate health and nutritional status, applying stratified, multistage, and probability sampling methods to obtain comprehensive data. This survey utilized a database of 80,312 participants from 8 cycles of NHANES in the United States from 2003 to 2018. After excluding participants with age < 20 years (*n* = 35,522) and missing or incomplete data on hyperlipidemia (*n* = 1871), CDAI (*n* = 7,890), and covariates (*n* = 7,403), we ultimately included 27,626 participants. Figure 1 shows a flow diagram of the entire sample selection process. The National Center for Health Statistics (NCHS) Ethics Review Committee approved the research, and all subjects signed an informed consent form.

### 2.2 Measurement of CDAI

During the NHANES survey, a professional statistician conducted two 24-h recall dietary interviews to collect information on the intake of nutritional antioxidants and other food components. The primary recollection interview was performed at the Mobile Screening Center, and the secondary recollection interview was obtained 3–10 days later through a telephone inquiry. Participants were asked to recall the specifics of all diets over 24 h, and dietary supplement intake, including dose, frequency, and duration, was determined (25). The Composite Dietary Antioxidant Index (CDAI) includes six dietary antioxidants: vitamins A, C, and E, zinc, selenium, and carotenoids. This dietary antioxidant access excludes antioxidants in nutritional supplements, medications, or drinking water. The CDAI was calculated by subtracting the mean and dividing by the standard deviation of an individual’s intake, and the results were as follows:

$\mathrm{CDAI}=\overset{6}{\underset{i=1}{\sum }}\frac{\mathrm{Individual}\;\mathrm{Intake}-\mathrm{Mean}}{SD}$.

### 2.3 Definition of hyperlipidemia

Relying on the National Cholesterol Education Program (NCEP) Adult Treatment Panel III (ATP-III), we estimated the Hyperlipidemia status. We defined Hyperlipidemia as (1) triglycerides (TG) ≥ 150 mg/dL (1.7 mmol/L), (2) total cholesterol (TC) ≥ 200 mg/dL (5.18 mmol/L), (3) low-density lipoprotein (LDL-C) ≥ 130 mg/dL (3.37 mmol/L), (4) high-density lipoprotein (HDL-C<), ≤40 mg/dL (1.04 mmol/L) for men or ≤ 50 mg/dL (1.30 mmol/L) for women, and (5) a participant that reported the use of lipid-lowering drugs was at the same time defined as having hyperlipidemia (3, 26–28). Hyperlipidemia was diagnosed if any of these five conditions were met.

### 2.4 Assessment of covariates

Based on previous studies and confounders, we selected the following covariates including age, sex (male/female), race (Mexican American/other Hispanic/non-Hispanic white/non-Hispanic black/other race), educational attainment (less than 9th grade, 9th–11th grade, high school graduation, some college graduation, and college and beyond), marital status (married, widowed, divorced, separated, unmarried, living with a partner), and household poverty-to-income ratio (PIR, <1.3, 1.3–3.5, ≥3.5); income-to-poverty ratios were calculated based on the US Department of Health and Human Services (DHHS) Family Income Poverty Guidelines (29). Smoking was categorized as (1) non-smoking (2) former tobacco (3) current smoking. Alcohol consumption was categorized as (1) no alcohol consumption, (2) previous alcohol consumption, (3) mild alcohol consumption, (4) moderate alcohol consumption, (5) heavy alcohol consumption. Body mass index (BMI) is weight (Kg) divided by the square of height (m). The glomerular filtration rate (eGFR) was calculated based on the serum creatinine equation (30). Energy, carbohydrate, total fat, and cholesterol intake were determined by averaging dietary recall over 2 days; coronary artery disease and lipid-lowering medications were obtained by a questionnaire that recorded whether participants were told by a physician that they had coronary artery disease and were taking lipid-lowering medications. The definition of diabetes was sufficient to satisfy any of the following: (1) self-reported physician diagnosis of diabetes mellitus, (2) current use of glucose-lowering medications or insulin injections, an (3) randomized Blood glucose ≥11.1 mmol/L, (4) Glycated hemoglobin (HbA1c) level ≥ 6.5%, (5) Fasting blood glucose (FPG) level ≥ 7.0 mmol/L, and (6) 2-h OGTT blood glucose level ≥ 11.1 mmol/L. The definition of hypertension can be satisfied with any one of the following: (1) self-reported high blood pressure, (2) patients taking antihypertensive medication, (3) mean systolic blood pressure (SBP) ≥140 mmHg and/or mean diastolic blood pressure (DBP) ≥90 mmHg; detailed measurement procedures for all covariates are available at www.cdc.gov/nchs/nhanes/index.htm.

### 2.5 Statistical analysis

All data in this study were statistically analyzed using the R language (version 4.3.1) and followed the guidelines established by the Centers for Disease Control and Prevention (CDC). We weighted the data in the data analysis, applying mean ± standard deviation for continuous variables and frequency (percentage) for categorical variables. Differences between categorical variables were analyzed using the chi-square test, while continuous variables conforming to normal distribution were tested with the weighted Student’s *t*-test; otherwise, they were tested with the Man-Whitney *u*-test. The correlation between CDAI and hyperlipidemia was studied using multifactor logistic regression modeling. CDAI was transformed into categorical variables by quartiles, and the linear trend test and *p*-value of the trend were calculated to determine the consistency of the relationship. Three models were constructed for this research: Model 1, a crude model without adjusting for any variables; Model 2, adjusted for sex, age, and race; and Model 3, which continued to change for education, marriage, PIR, eGFR, BMI, energy, carbohydrates, total fat, cholesterol, smoking, alcohol, hypertension, diabetes mellitus, coronary artery disease, and lipid-lowering medications based on Model 2. Smoothed curve fitting was applied to explore further whether there was a linear relationship between CDAI and hyperlipidemia. In addition, to investigate whether there were interactions and stability between subgroups, we performed subgroup-stratified analyses for age, gender, race, education level, marriage, smoking status, alcohol consumption, BMI, hypertension, and diabetes mellitus. A *p*-value of below 0.05 was recognized as statistically significant.

## 3 Results

### 3.1 Characteristics of participants

This research included 27,626 participants, with a mean age of 47.42 ± 0.24 years, of which 51.23% were female and 48.77% were male. Baseline characteristics of participants by CDAI quartiles are shown in Table 1, differences between quartiles were statistically significant (*p* < 0.05) for age, sex, race, education, marriage, PIR, smoking, alcohol consumption, BMI, eGFR, energy, carbohydrates, total fat, cholesterol, hypertension, diabetes mellitus, coronary artery disease, and use of lipid-lowering drugs. Compared to Q1, Q4 tended to be younger, male, non-Hispanic white, with lower BMI, better economic standard, and higher education status. Non-smoking and mild alcohol consumption had greater levels of CDAI. The higher the CDAI level, the more energy, carbohydrates, total fat, and cholesterol they consume. Hypertension, diabetes mellitus, coronary heart disease, and hyperlipidemia decreased with increasing CDAI.

**Table 1:** The characteristics of participants.

Variable	Total	Q1 (−8.664, −2.209)	Q2 (−2.209, −0.002)	Q3 (−0.002, 2.774)	Q4 (2.774, 124.284)	*p*-value
*N*	27,626	6,907	6,906	6,907	6,906	
Age, year	47.42 ± 0.24	47.88 ± 0.36	48.10 ± 0.34	47.35 ± 0.38	46.56 ± 0.35	0.001
Gender, *n* (%)						<0.0001
Female	14,008 (51.23)	3,792 (57.88)	3,471 (51.40)	3,416 (49.84)	3,329 (47.33)	
Male	13,618 (48.77)	3,115 (42.12)	3,435 (48.60)	3,491 (50.16)	3,577 (52.67)	
Race, *n* (%)						<0.0001
Mexican American	4,231 (8.01)	1,074 (8.06)	1,135 (8.70)	1,040 (7.89)	982 (7.50)	
Non-Hispanic Black	5,558 (10.22)	1,741 (14.35)	1,398 (11.06)	1,214 (8.39)	1,205 (8.09)	
Non-Hispanic White	13,160 (70.38)	2,959 (65.68)	3,238 (68.75)	3,449 (72.52)	3,514 (73.30)	
Other race	2,423 (6.70)	498 (6.51)	586 (7.05)	637 (6.41)	702 (6.81)	
Others	2,254 (4.70)	635 (5.40)	549 (4.44)	567 (4.78)	503 (4.30)	
Education, *n* (%)						<0.0001
9–11th grade	3,702 (9.76)	1,218 (14.87)	955 (10.00)	815 (8.41)	714 (6.94)	
College graduate or above	6,635 (30.26)	932 (17.00)	1,474 (26.45)	1,926 (34.22)	2,303 (39.83)	
High school graduate	6,409 (23.38)	1,813 (29.28)	1,670 (25.17)	1,535 (21.87)	1,391 (18.79)	
Less than 9th grade	2,543 (4.49)	1,012 (7.36)	689 (5.09)	507 (3.86)	335 (2.38)	
Some college	8,337 (32.12)	1,932 (31.49)	2,118 (33.29)	2,124 (31.64)	2,163 (32.06)	
Marital status, *n* (%)						<0.0001
Divorced	3,050 (10.52)	882 (12.70)	749 (10.31)	701 (9.79)	718 (9.73)	
Living with partner	2,108 (7.67)	539 (8.59)	517 (8.44)	506 (7.08)	546 (6.87)	
Married	14,731 (56.21)	3,312 (48.69)	3,708 (55.97)	3,875 (58.99)	3,836 (59.53)	
Never married	4,691 (17.87)	1,220 (19.69)	1,133 (17.33)	1,118 (16.66)	1,220 (18.11)	
Separated	848 (2.15)	262 (3.16)	220 (1.96)	190 (2.04)	176 (1.64)	
Widowed	2,198 (5.57)	692 (7.17)	579 (5.99)	517 (5.44)	410 (4.11)	
PIR						<0.0001
<1.30	8,094 (20.25)	2,653 (28.63)	2,077 (21.98)	1,734 (17.07)	1,630 (15.41)	
1.30–3.50	10,537 (35.16)	2,734 (38.80)	2,703 (36.25)	2,616 (33.93)	2,484 (32.63)	
≥3.50	8,995 (44.59)	1,520 (32.57)	2,126 (41.77)	2,557 (49.00)	2,792 (51.96)	
Smoking status, *n* (%)						<0.0001
Former	7,085 (25.20)	1,648 (21.57)	1,824 (25.33)	1,808 (26.29)	1,805 (26.82)	
Never	14,972 (54.80)	3,376 (48.47)	3,736 (53.90)	3,871 (56.40)	3,989 (58.87)	
Now	5,569 (20.00)	1,883 (29.96)	1,346 (20.76)	1,228 (17.31)	1,112 (14.30)	
Alcohol use, *n* (%)						<0.0001
Former	4,807 (13.85)	1,453 (16.96)	1,256 (14.40)	1,090 (12.60)	1,008 (12.21)	
Heavy	5,331 (20.95)	1,366 (23.09)	1,333 (21.73)	1,278 (19.91)	1,354 (19.66)	
Mild	9,497 (36.80)	1,955 (29.90)	2,394 (36.38)	2,541 (37.82)	2,607 (41.47)	
Moderate	4,294 (17.72)	993 (16.73)	1,002 (16.57)	1,144 (19.22)	1,155 (18.00)	
Never	3,697 (10.68)	1,140 (13.31)	921 (10.92)	854 (10.46)	782 (8.67)	
BMI, kg/m^2^	28.97 ± 0.09	29.25 ± 0.12	29.23 ± 0.13	28.93 ± 0.14	28.58 ± 0.15	0.001
eGFR, mL/min/1.73 m2	93.63 ± 0.32	93.04 ± 0.45	92.66 ± 0.45	93.80 ± 0.49	94.77 ± 0.44	<0.001
Energy, kcal	2117.91 ± 8.49	1442.51 ± 10.46	1898.05 ± 10.80	2226.76 ± 11.80	2716.42 ± 17.52	<0.0001
Carbohydrate, g	252.27 ± 1.06	179.44 ± 1.71	227.01 ± 1.64	263.32 ± 1.83	318.78 ± 2.27	<0.0001
Total fat, g	81.79 ± 0.42	52.32 ± 0.43	72.58 ± 0.52	87.10 ± 0.61	107.03 ± 0.88	<0.0001
Cholesterol, mg	292.17 ± 1.87	183.38 ± 2.14	261.21 ± 2.61	311.55 ± 3.55	383.03 ± 4.41	<0.0001
Hypertension						<0.001
No	15,695 (62.47)	3,617 (60.15)	3,798 (60.08)	4,061 (64.00)	4,219 (64.81)	
Yes	11,931 (37.53)	3,290 (39.85)	3,108 (39.92)	2,846 (36.00)	2,687 (35.19)	
CHD						0.001
No	26,415 (96.37)	6,538 (95.67)	6,581 (95.83)	6,625 (96.54)	6,671 (97.19)	
Yes	1,211 (3.63)	369 (4.33)	325 (4.17)	282 (3.46)	235 (2.81)	
DM						<0.0001
No	22,650 (86.78)	5,426 (84.59)	5,546 (84.47)	5,778 (88.33)	5,900 (88.93)	
Yes	4,976 (13.22)	1,481 (15.41)	1,360 (15.53)	1,129 (11.67)	1,006 (11.07)	
AntiHyperlipidemic						<0.001
No	21,984 (82.28)	5,387 (82.05)	5,360 (79.89)	5,520 (82.78)	5,717 (83.99)	
Yes	5,642 (17.72)	1,520 (17.95)	1,546 (20.11)	1,387 (17.22)	1,189 (16.01)	
Hyperlipidemia						<0.0001
No	7,623 (28.91)	1,716 (26.27)	1,787 (27.11)	1,939 (29.38)	2,181 (32.00)	
Yes	20,003 (71.09)	5,191 (73.73)	5,119 (72.89)	4,968 (70.62)	4,725 (68.00)	
CDAI	1.02 ± 0.06	−3.74 ± 0.03	−1.10 ± 0.01	1.25 ± 0.01	6.24 ± 0.07	<0.0001
Vitamin A, ug	650.77 ± 6.49	287.55 ± 3.57	480.49 ± 4.12	654.27 ± 5.15	1069.26 ± 14.72	<0.0001
Vitamin C, mg	83.00 ± 0.94	35.67 ± 0.55	60.93 ± 0.83	85.27 ± 1.10	135.69 ± 1.75	<0.0001
Vitamin E, mg	8.42 ± 0.08	4.17 ± 0.04	6.36 ± 0.05	8.51 ± 0.07	13.33 ± 0.14	<0.0001
Zinc, mg	11.79 ± 0.08	6.76 ± 0.05	9.80 ± 0.07	12.19 ± 0.09	16.92 ± 0.18	<0.0001
Selenium, ug	114.08 ± 0.59	70.51 ± 0.54	99.12 ± 0.56	119.80 ± 0.67	154.57 ± 1.26	<0.0001
Carotenoid, ug	9990.59 ± 136.86	3558.74 ± 62.66	6485.76 ± 85.01	9735.40 ± 112.53	18118.56 ± 276.35	<0.0001

PIR, Income to poverty ratio; BMI, body mass index; eGFR, estimated glomerular filtration rate; CHD, coronary heart disease; DM, diabetes mellitus; CDAI, composite dietary antioxidant index.

### 3.2 Relationship between CDAI and hyperlipidemia

**Figure 2:**
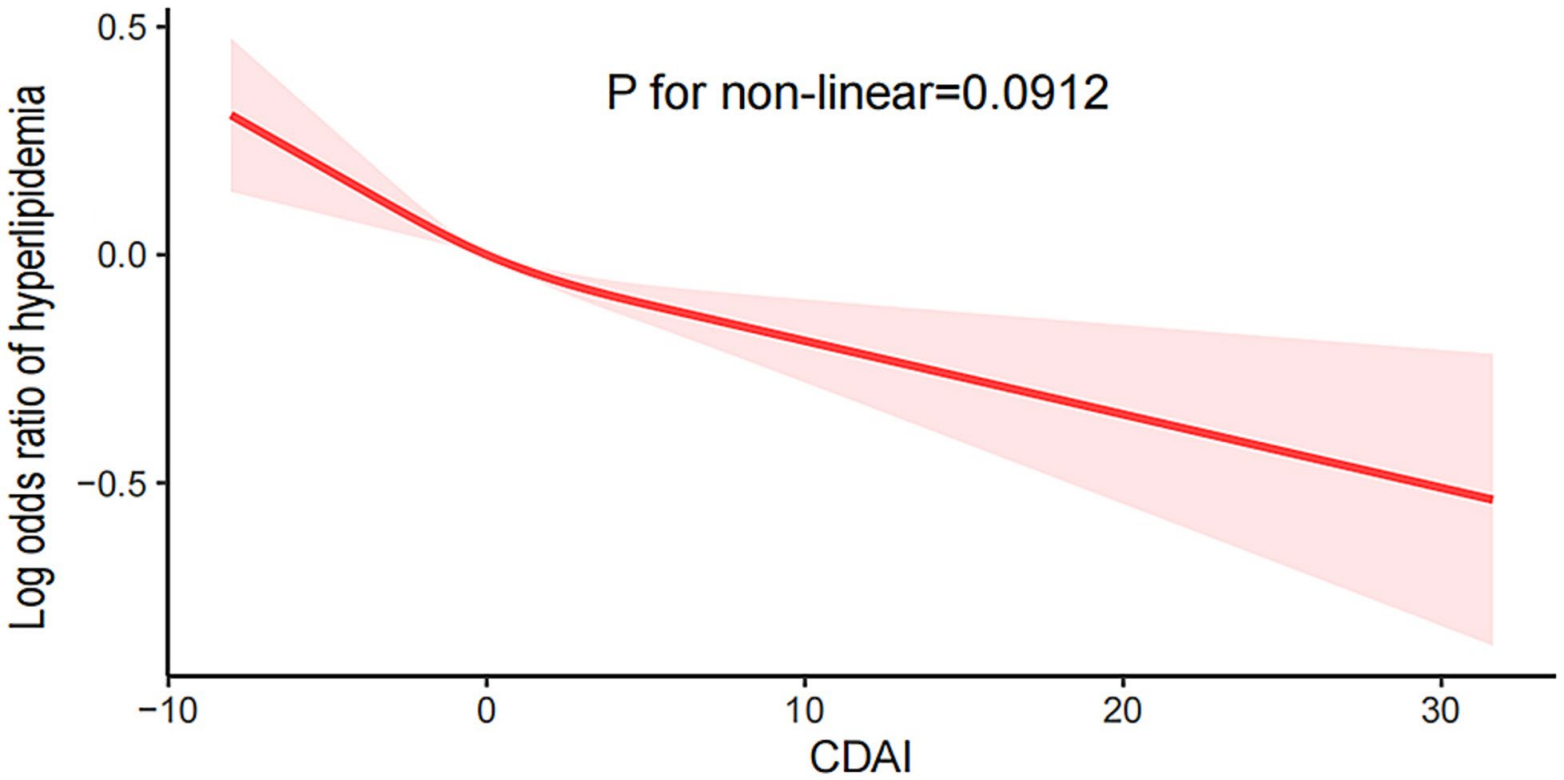
The smooth curve fitting analysis of CDAI and hyperlipidemia.

The multivariate logistic regression model, as shown in Table 2, demonstrates the correlation between CDAI and the risk of hyperlipidemia. In model 3, CDAI scores were negatively associated with the risk of developing hyperlipidemia (OR 0.98, 95% CI 0.96–0.99). This negative correlation was more pronounced in participants with a lower predominance ratio of hyperlipidemia than in Q1 according to quartile stratification in Q2 (OR 0.91, 95% CI 0.78–1.06), Q3 (OR 0.85, 95% CI 0.73–1.00), and Q4 (OR 0.77, 95% CI 0.64–0.94) and was also confirmed by a test of trend in each model using Q1 as control this (*p* < 0.05). We also used smoothed curve fitting to assess the association between the two; as shown in Figure 2, the correlation between CDAI and hyperlipidemia was negative and linear (p for non-linear = 0.0912).

**Table 2:** The association between CDAI and hyperlipidemia.

Character	Model 1		Model 2		Model 3	
	95%CI	*p*	95%CI	*p*	95%CI	*p*
CDAI	0.97 (0.97, 0.98)	<0.0001	0.97 (0.97, 0.98)	<0.0001	0.98 (0.96, 0.99)	0.01
**CDAIQ**						
Q1	1 (ref)		1 (ref)		1 (ref)	
Q2	0.96 (0.84, 1.09)	0.50	0.93 (0.81, 1.06)	0.28	0.91 (0.78, 1.06)	0.21
Q3	0.86 (0.76, 0.97)	0.01	0.84 (0.74, 0.95)	0.01	0.85 (0.73, 1.00)	0.05
Q4	0.76 (0.67, 0.86)	<0.0001	0.75 (0.66, 0.85)	<0.0001	0.77 (0.64, 0.94)	0.01
*p* for trend	<0.0001		<0.0001		0.01	

Model 1: non-adjusted.

Model 2: adjusted age, gender, race.

Model 3: adjusted age, gender, race, education, marital, PIR, smoke, alcohol, eGFR, BMI, energy, carbohydrate, total, cholesterol, DM, hypertension, CHD, antiHyperlipidemic.

### 3.3 Subgroup analysis

**Figure 3:**
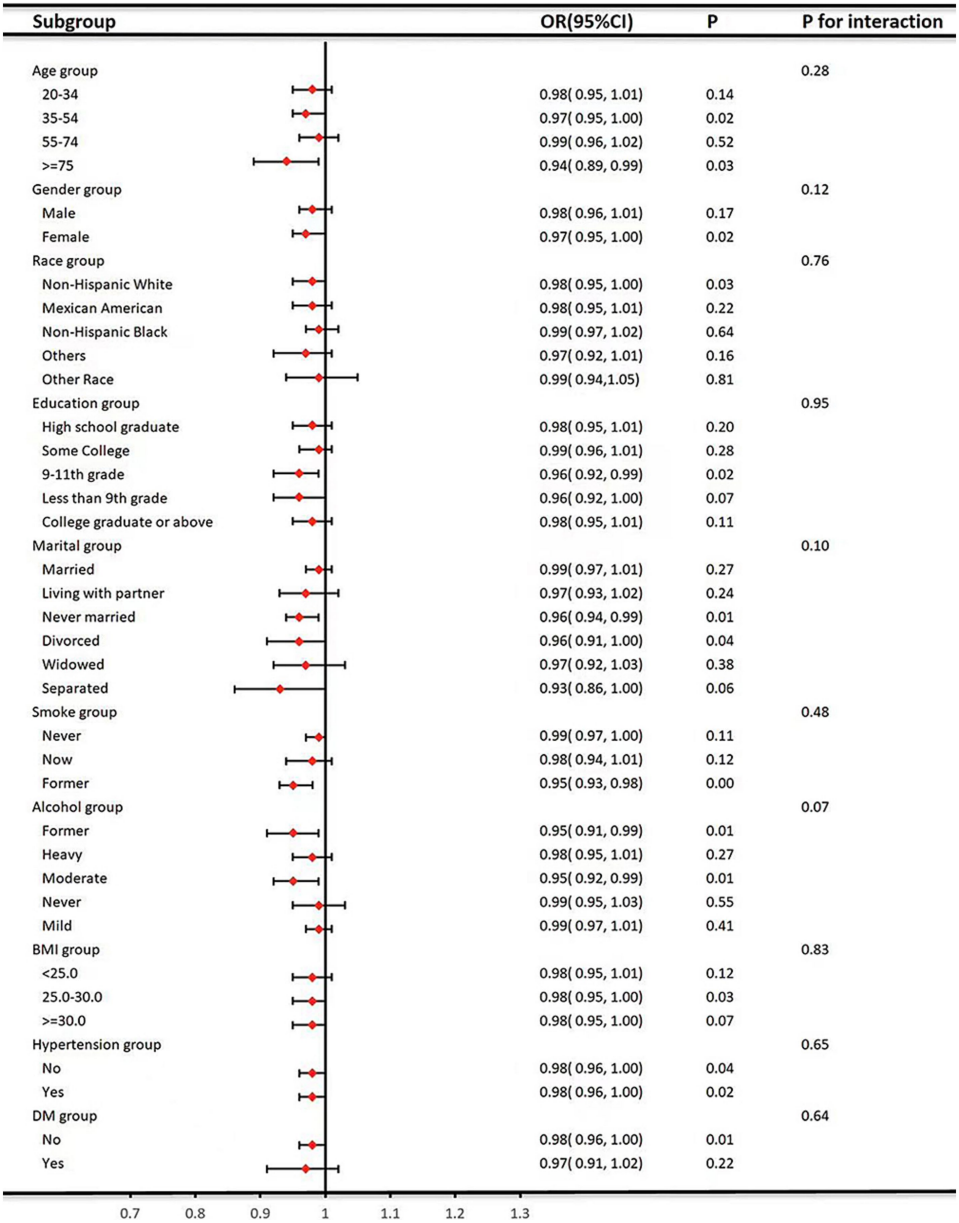
Subgroup analysis for the association between CDAI and hyperlipidemia.

As shown in Figure 3, we stratified age, gender, race, education level, marriage, smoking status, alcohol consumption, BMI, hypertension, and diabetes to explore the stability of the relationship between CDAI and hyperlipidemia and the presence or absence of interaction. In subgroup analyses, no significant interactions were found between CDAI and these stratified variables (*p* > 0.05), and the relationship was very stable.

## 4 Discussion

The study analyzed the most representative US population data and, after adjusting for several covariates, found that CDAI was negatively associated with the prevalence of hyperlipidemia in the population, which confirms our hypothesis. These results suggest that appropriate dietary modification and increased antioxidant intake can help prevent and treat hyperlipidemia. Although previous studies have demonstrated the effect of dietary antioxidants such as VA, VE, and zinc on hyperlipidemia, most of them applied a single class of antioxidants for correlation analysis and did not consider them in combination. However, the intake of a single antioxidant is difficult to achieve in the daily diet, and it is more likely that a wide range of foods and a wide range of antioxidants will be consumed. In addition, there is no single antioxidant component in foods that are rich in multiple antioxidants. Therefore, there is a need to investigate the relationship between combined dietary antioxidant intake and hyperlipidemia.

Oxidative stress plays a vital role in hyperlipidemia. Wu et al. (31) found that the substrates of *G. frondosa* decreased glutathione (GSH) and catalase activity, increased GSH peroxidase activity, inhibited oxidative stress, and increased bile acid synthesis to improve hypercholesterolemia. Feng et al. (32) administered hawthorn fructofuranic acid (HFA) to rats. Found that activation of Nrf2/HO-1 signaling pathway could inhibit oleic acid (OA)-induced oxidative damage in a hepatocellular carcinoma cell line (HepG2), which in turn reduced oxidative stress damage in rats, effectively reduced triglyceride and cholesterol levels, attenuated hepatic steatosis and intervened in hyperlipidemia. Pretreatment with *M. charantia* polysaccharides for 25 days in a rat model was found to inhibit oxidative stress via nuclear factor κB (NF-kB), lowering lipid indices, and consequently ameliorating hyperlipidemia (33). Ji et al. (34) further found that consumption of propolis by rabbits with hypercholesterolemia lowered the lipid levels and enhanced antioxidant activity by a mechanism which may be the inhibition of the TLR4-mediated NF- κB signaling pathway. Puerarin was found to reduce ROS production and increase antioxidant capacity by modulating the phosphorylated Jun N-terminal kinase (JNK)/phosphorylated c-Jun protein/cholesterol 7a-hydroxylase (CYP7A1) pathway in mouse liver, which in turn inhibited hyperlipidemia (35). Fan et al. (36) used baicalein to intervene in hyperlipidemic rats and showed results of enhanced activation of PI3K/AKT and activation of Nrf2, thereby increasing the expression of HO-1 and NQO1, inhibiting oxidative stress and preventing hyperlipidemia. Honey pretreatment given to rats before feeding them a high-fat diet was found to be able to attenuate oxidative stress and exert anti-hyperlipidemia effects more significantly by inhibiting 3-hydroxy-3-methylglutaryl coenzyme A (HMG-CoA) reductase (37). Antunes et al. (38) experimented with different combinations of phenolic compounds from purple grape bark, both *in vitro* and *in vivo*, and all of the results showed an antioxidant effect, thereby reducing hypercholesterolemia. Administration of p-coumaric acid (p-CA) in mouse experiments revealed an increase in the expression of Nrf2, HO-1, glutathione peroxidase (Gpx), and superoxide dismutase (SOD-1), which in turn exerted an antioxidant and potentially therapeutic effect in the treatment of hyperlipidemia (39). In conclusion, inhibition of oxidative stress, which can treat hyperlipidemia, is the same as the conclusions of cross-sectional studies on the relationship between CDAI and hyperlipidemia.

This study also found that the prevalence of hypertension, diabetes mellitus, and coronary heart disease decreased progressively with increasing CDAI. Previous analyses of the NHANES database found that the prevalence of hypertension, coronary heart disease, and diabetes mellitus were negatively correlated with CDAI (18, 23, 40), which is consistent with our findings. Control variables such as energy, carbohydrates, total fat, and cholesterol, which may affect the results of the study, were added to the previous ones. In addition, instead of using the traditional Dietary Antioxidant Quality Score (DAQS) for assessing dietary antioxidant indices in this study, the more accurate CDAI was used for correlation analyses because of its applied advantages and validity proved in epidemiological studies.

Diet is essential in treating hyperlipidemia by containing antioxidants that reduce oxidative stress damage and promote health (41). Antioxidants are biologically active compounds that neutralize free radicals and reduce oxidative damage (42). Although there are no studies on the relationship between CDAI and hyperlipidemia, the use of dietary antioxidant properties to intervene in hyperlipidemia has become a hot research topic. The vitamins (A, D, and E) and carotenoids in CDAI are fat-soluble and vitamin C is water-soluble, which is relevant for absorption, transport and action. Feeding a VA-rich (52 mg/kg) diet for 20 weeks in hypercholesterolemic obese rats increased plasma lecithin cholesterol acyltransferase activity and expression of ATP-binding cassette transporter protein A1, which, in turn, led to normalization of plasma HDL-C levels (43). Vitamin C (VC) is an essential nutrient for humans and the effect of administration is highly dose dependent (44), maximizing neutrophil concentration, reducing ROS production and inhibiting LDL-C oxidation through dietary intake (45, 46). Kumar et al. (47) administered VC (0.5 g/kg) once daily for 30 days in a male rat model of hyperlipidemia. It showed a decrease in triglycerides, cholesterol, ROS, malondialdehyde (MDA), and reduced GSH, the mechanism of which may be to prevent hyperlipidemia by inhibiting oxidative stress and maintaining redox balance. In a randomized double-blind crossover study, VC (1,000 mg) in combination with dietary nitrates given to untreated hypercholesterolemic subjects for 4 weeks was found to reduce oxidized low-density lipoprotein (oxLDL), triglycerides and inhibit oxidative stress by increasing the total plasma NO metabolites (48). Selenium was able to maintain the anti-oxidative stress function of glutathione peroxidation and selenoprotein P (49). Guo et al. (50) fed selenium nanoparticles (SeNPs) 50 μg/d in apolipoprotein E-deficient (ApoE) mice, and showed that it was able to alleviate hyperlipidemia in ApoE mice by regulating cholesterol metabolism through antioxidant selenoenzymes/selenoproteins and reducing oxidative stress, and additionally metabolomics selenium-enriched kiwifruit was also found to be able to treat hyperlipidemia (51). Carotenoids (Crt) act as antioxidants in lipid-rich environments (52), mainly through (i) electron transfer between free radicals (R-) and Crt, leading to the formation of Crt radical cation (Crt-+) or Crt radical anion (Crt-); and (ii) radical adducts (R-), leading to the formation of Crt radical cation (Crt-+) or Crt radical anion (Crt-). (-); (ii) radical adduct formation (RCrt-); and (iii) hydrogen atom transfer leading to neutral Crt radicals (Crt-) three pathways to scavenge free radicals, which can also effectively scavenge ROS and play an antioxidant role (53). Lycopene, a water-soluble carotenoid, intervenes in hyperlipidemia by down-regulating oxidative stress induced by the preprotein convertase *Bacillus subtilis* protease/kexin type 9 (PCSK-9) targeting lipopolysaccharide (LPS) (54). This study concluded that the difference between single serum antioxidant and hyperlipidemia was statistically significant, and the more intake, the greater the CDAI and the lower the prevalence of hyperlipidemia, which is in line with the results of the above study.

The following four limitations exist in this research. First, the study was a cross-sectional analysis, and we could not determine a causal relationship between CDAI and hyperlipidemia. Second, since the population studied was Americans, special people such as minors were omitted. Third, some unknown covariates may affect the relationship between CDAI and the risk of hyperlipidemia. Fourth, CDAI data are self-reported and will be subject to recall bias.

## 5 Conclusion

There is a linear negative association between CDAI and the risk of hyperlipidemia. Therefore, intake of foods rich in CDAI components, which are mainly rich in VC and VE, may reduce the risk of hyperlipidemia. In the future, prospective studies and basic experiments are needed to further explore the mechanism of action, tap into the diet of each component of CDAI to exclude the effect of individual variability, and additionally explore the relationship between more antioxidants in the diet and hyperlipidemia to guide the diet. Living with balanced nutrition, reasonable diet, strengthening physical exercise, improving body metabolism, strengthening hyperlipidemia prevention and reducing the occurrence of the disease.

## Data availability statement

The raw data supporting the conclusions of this article will be made available by the authors, without undue reservation.

## Ethics statement

The studies involving humans were approved by The Research Ethics Review Board at the National Center for Health Statistics (NCHS). The patients/participants provided their written informed consent to participate in this study. The studies were conducted in accordance with the local legislation and institutional requirements. The participants provided their written informed consent to participate in this study.

## Author contributions

HZ: Data curation, Formal analysis, Methodology, Software, Validation, Writing – original draft, Writing – review & editing. TL: Conceptualization, Data curation, Formal analysis, Methodology, Writing – review & editing. JL: Conceptualization, Methodology, Visualization, Writing – review & editing. DZ: Formal analysis, Software, Visualization, Writing – review & editing. JY: Conceptualization, Methodology, Validation, Visualization, Writing – review & editing. XZ: Data curation, Supervision, Visualization, Writing – review & editing.
